# Intrathoracic myolipoma from parietal pleura with oestrogen and progesterone receptor

**DOI:** 10.1002/rcr2.853

**Published:** 2021-09-21

**Authors:** Jyh Shinn Teh, Ching‐Yao Weng, Ying‐Yueh Chang, Yi‐Chen Yeh, Chien‐Sheng Huang

**Affiliations:** ^1^ Department of Surgery Taipei Veterans General Hospital Taipei Taiwan; ^2^ Department of Radiology Taipei Veterans General Hospital Taipei Taiwan; ^3^ Department of Pathology Taipei Veterans General Hospital Taipei Taiwan; ^4^ Division of Thoracic Surgery, Department of Surgery Taipei Veterans General Hospital Taipei Taiwan; ^5^ Department of Surgery, School of Medicine National Yang‐Ming Chiao Tung University Taipei Taiwan

**Keywords:** histology, imaging/CT MRI, myolipoma, pleural tumour

## Abstract

Myolipoma is a rare benign lipomatous soft tissue neoplasm, occurring most frequently in adults in the abdomen, pelvis or retroperitoneum. We presented a case of a 39‐year‐old female with an epipleural lesion at the left paraspinal region identified using computed tomography. Magnetic resonance imaging revealed a fat‐containing lesion attached to the pleura over the left paraspinal region. Surgical resection was performed, and histopathological analysis revealed a tumour composed of interlacing bundles of neoplastic smooth muscle cells with low cellularity and rare mitotic figures intermixing with mature adipocytes. The presence of oestrogen and progesterone receptors in smooth muscle cells was noted. The mass was reported as myolipoma. To the best of our knowledge, this case is the first description of an intrathoracic myolipoma.

## Graphical abstract

Myolipoma should be considered as one of the differential diagnosis in intrathoracic tumour, with its benign clinical course with simple surgical excision. The presence of oestrogen and progesterone receptor in myolipoma was noted in our case, which was also noted in some of the reported myolipoma cases. Symptoms disclosed after pregnancy suggested the promotion of tumour growth by oestrogen and progesterone.

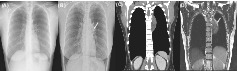

## INTRODUCTION

Myolipoma is an extremely rare benign soft tissue tumour composed of a mixed proliferation of adipose and smooth muscle tissues. It presents as a large, painless and palpable mass in most patients. The tumour is benign with no reported cases of recurrence nor metastasis after surgical resection. Most cases of this tumour have been reported in the retroperitoneum, pelvis and abdomen. There are fewer reports of the tumour in the round ligament, spinal cord, eyelid, subcutaneous tissue and pericardium. We present the first case of an intrathoracic myolipoma described in literature.

## CASE REPORT

**FIGURE 1:**
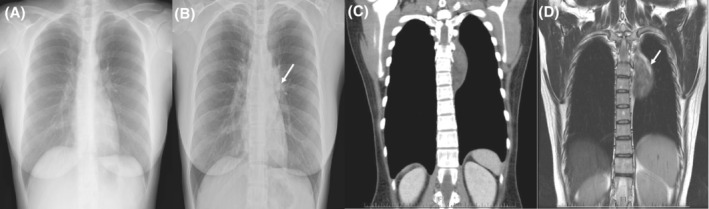
Chest x‐ray. (A) No mediastinum mass noted 4 years prior to visit. (B) A faint bulging mass (white arrow) was noted at the posterior mediastinum, left paraspinal region. (C) Computed tomography of the intrathoracic tumour: heterogeneous density with contrast‐enhanced fat‐containing lesion at the left paraspinal region. (D) Magnetic resonance imaging of the intrathoracic tumour, T2‐weighted image (sagittal view): heterogeneous intensity fat‐containing lesion at the paraspinal region (arrow)

**FIGURE 2:**
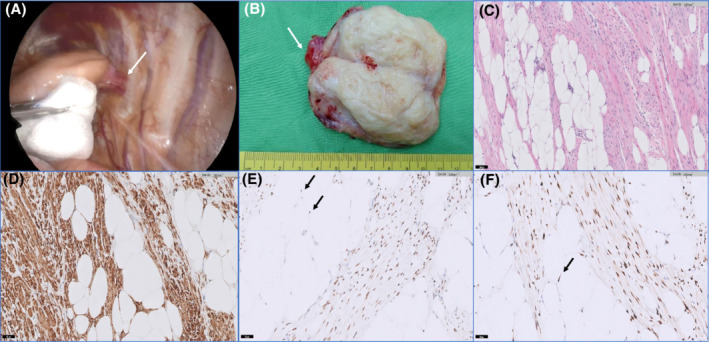
(A) Intraoperative finding: an oval shaped, fat‐containing, pedunculated tumour is attached to pleural tissue over the posterior fourth intercostal space (white arrow: stalk). (B) Gross appearance of the intrathoracic tumour: the cut surface of the tumour is yellow with small nodules of firm white tissue corresponding to smooth muscle (white arrow: stalk). (C) Histological appearance of the intrathoracic tumour: admixture of smooth muscle and adipocytes (magnification: 100×). (D) Smooth muscle cells are immunoreactive with actin stain (magnification: 200×). (E) Smooth muscle and mature adipocytes nuclear are positive for oestrogen receptor (black arrows: mature adipocytes; magnification 200×). (F) Smooth muscle and mature adipocytes are positive for progesterone receptor (black arrows: mature adipocytes; magnification: 200×)

We present a case of a 39‐year‐old female who reported left upper back soreness over the past 6 years (since her first pregnancy). A chest x‐ray revealed a faint bulging mass over the posterior mediastinum, left paraspinal region, which was not found in an x‐ray taken 4 years previously. Computed tomography (CT) disclosed an epipleural, heterogeneous, contrast‐enhancing,, fat‐containing lesion at the left paraspinal region. Magnetic resonance imaging (MRI) showed an oval, fat‐containing, contrast‐enhancing mass in the left paraspinal region attached to the pleura (Figure 1). Thoracoscopic tumour removal was performed in September 2020. An oval, fat‐containing, pedunculated tumour over the posterior fourth intercostal space was noted. Surgical resection by way of disconnection of the tumour peduncle and the parietal pleura was performed. The mass measured approximately 8 × 5.5 × 3.5 cm in size and was made up of soft yellow tissue with firm white tissue. Histopathological analysis revealed the tumour was composed of smooth muscle cells with low cellularity and rare mitotic figures intermixed with mature adipocytes (Figure 2). Immunohistochemical analysis was positive for smooth muscle actin (SMA), HHF‐35, desmin and h‐caldesmon, and negative for melanoma markers (HMB45 and Melan‐A), MDM2, CDK4, CD34 and Sat6, which suggested myolipoma. The presence of oestrogen and progesterone receptors was noted, strongly in smooth muscle cells and with low positivity in mature adipocytes. Follow‐up occurred 9 months later (July 2021) and showed no significant evidence of recurrence or metastasis. To the best of our knowledge, this is the first description of an intrathoracic myolipoma.

## DISCUSSION

Myolipoma, a rare benign tumour that was first described by Meis and Enzinger in 1991, was mostly reported in the retroperitoneum, pelvis and abdomen.^1^ There are fewer reports of the tumour in the round ligament, spinal cord, eyelid, tongue base, mesentery and pericardium. Myolipoma occurs frequently in adults, with a slight predilection for women, and presents clinically as an enlarged painless mass or as a variety of symptoms according to anatomic site. A CT scan may reveal images similar to subcutaneous fat or a heterogeneous mass with muscle and fat tissue. An MRI may reveal the lesion with intermediate to high signal intensity in a T2‐weighted image, and with intermediate signal intensity of smooth muscle in a T1‐weighted image.^2^ Liposarcoma, angiomyolipoma and myelolipoma^3^ have similar radiological features that should be considered as part of a differential diagnosis; hence, histopathology is essential for the diagnosis of myolipoma.

Myolipoma has been described as a completely or partially encapsulated mass. The presence of a stalk supporting the tumour was noted in the current case, but has been rarely mentioned in other published cases. Histologically, it consisted of mature adipocytes and well‐differentiated smooth muscle. Differential diagnosis includes lipoleiomyosarcoma, well‐differentiated liposarcoma, dedifferentiated liposarcoma, spindle cell lipoma, mammary‐type myofibroblastoma and fat‐forming solitary fibrous tumour. In the present case, the immunohistochemical stains were positive for SMA, HHF‐35, desmin and h‐caldesmon, and negative for melanoma markers (HMB45 and Melan‐A), MDM2, CDK4, CD34 and Sat6. Differential diagnosis of myolipoma using immunohistochemical staining to distinguish other neoplasms is summarized in Table 1. HMGA2 immunohistochemical staining was not available; however, it is worth noting that a positive stain for HMGA2 is not a required marker for the diagnosis of myolipoma. However, it has been reported that approximately 60% of myolipoma express HMGA2, which indicates that approximately 40% of myolipomas are negative. HMGA2 is widely expressed in other lipomatous tumours, such as lipoma (86%), well‐differentiated liposarcoma (86%) and dedifferentiated liposarcoma (67%).^4^ Due to the low sensitivity and specificity of HMGA2, it may not be useful in the differential diagnosis of myolipoma.

**TABLE 1:** Pathological differential diagnosis of myolipoma

Tumour	Pathological characteristic	Immunohistochemical	
		Positive	Negative
Myolipoma	Absence of mitoses, no atypia	SMA Desmin HMGA2	HMB‐45 MDM2 CK4
Spindle cell lipoma	Admixture of adipocytes, short spindle cells and ropey collagen bundles	CD34	SMA Desmin
Mammary‐type myofibroblastoma	Different morphology of spindle cell	Desmin CD34	
Lipoleiomyosarcoma	Lipoblasts or atypical cells with hyperchromatic nuclei in irregular fibrous septa	MDM2 CDK4	
Well‐differentiated liposarcoma	Adipocytic nuclear atypia, with hyperchromatic stromal cells	MDM2 CDK4	HMB‐45
Dedifferentiated liposarcoma	Nuclear atypia, high mitotic activity	HMGA2 MDM2 CDK4	
Fat‐forming solitary fibrous tumour	Admixture of fibroblastic spindle cells and fat cells	CD34 Stat6	
Angiomyolipoma	Consist of mature fat cells, smooth muscle cells and thick‐walled vessels	HMB‐45 Melan‐A	
Myelolipoma	Consist of mature adipocyte and haematopoietic tissue		

Abbreviation: SMA, smooth muscle actin.

Consistent with the current case, the presence of both oestrogen and progesterone receptors was reported in myolipomas of the broad ligament and pericardium. Symptoms were first disclosed following the patient's first pregnancy. Rapid tumour growth occurred in the past 4 years, in which a second pregnancy occurred. It has been suggested that the growth of the myolipoma could be affected by pregnancy‐related hormones^5^; however, further studies are required to confirm and expand on this suggestion.

Myolipoma presents with a benign clinical course, with surgical excision as a curative treatment. To date, there have been no reports of recurrence, metastasis or malignancy. In summary, myolipoma should be considered as a differential diagnosis of fat‐containing intrathoracic masses in order to make an accurate diagnosis. Specific pathological and immunohistochemical characteristics of the tumour, including a positive stain for SMA, desmin and/or HMGA2, as well as a negative stain for HMB‐45 and MDM2, are helpful in the diagnosis of myolipoma.

## CONFLICT OF INTEREST

None declared.

## ETHICS STATEMENT

The authors declare that appropriate written informed consent was obtained for publication of this case report and accompanying images.
